# Model-based analysis of an adaptive evolution experiment with *Escherichia coli* in a pyruvate limited continuous culture with glycerol

**DOI:** 10.1186/1687-4153-2012-14

**Published:** 2012-10-03

**Authors:** Ronny Feuer, Katrin Gottlieb, Gero Viertel, Johannes Klotz, Steffen Schober, Martin Bossert, Oliver Sawodny, Georg Sprenger, Michael Ederer

**Affiliations:** 1 Institute for System Dynamics, University of Stuttgart, Pfaffenwaldring 9, 70569 Stuttgart, Germany; 2 Institute of Microbiology, University of Stuttgart, Stuttgart, Germany; 3 Institute of Communications Engineering, University of Ulm, Ulm, Germany

## Abstract

Bacterial strains that were genetically blocked in important metabolic pathways and grown under selective conditions underwent a process of adaptive evolution: certain pathways may have been deregulated and therefore allowed for the circumvention of the given block. A block of endogenous pyruvate synthesis from glycerol was realized by a knockout of pyruvate kinase and phosphoenolpyruvate carboxylase in *E. coli*. The resulting mutant strain was able to grow on a medium containing glycerol and lactate, which served as an exogenous pyruvate source. Heterologous expression of a pyruvate carboxylase gene from *Corynebacterium glutamicum* was used for anaplerosis of the TCA cycle. Selective conditions were controlled in a continuous culture with limited lactate feed and an excess of glycerol feed. After 200–300 generations pyruvate-prototrophic mutants were isolated. The genomic analysis of an evolved strain revealed that the genotypic basis for the regained pyruvate-prototrophy was not obvious. A constraint-based model of the metabolism was employed to compute all possible detours around the given metabolic block by solving a hierarchy of linear programming problems. The regulatory network was expected to be responsible for the adaptation process. Hence, a Boolean model of the transcription factor network was connected to the metabolic model. Our model analysis only showed a marginal impact of transcriptional control on the biomass yield on substrate which is a key variable in the selection process. In our experiment, microarray analysis confirmed that transcriptional control probably played a minor role in the deregulation of the alternative pathways for the circumvention of the block.

## Introduction

Since the long term evolution experiment of Lenski et al. [1], laboratory evolution has attracted much attention [2]. They demonstrated the adaptive behavior of mircoorganisms through shaking flask experiments with regular transfer in fresh culture media [1]. Already, Hoefle et al. [3] reported the presence of selective pressure in chemostat experiments. In the fermentation process, the adaptive evolution of the organisms occurs through random genetic mutation and controlled selection [4]. This process exhibits considerable potential for the design of industrial production strains [5]. Small product yields, slow growth, evolutive instability of mutated strains or toxicity of byproducts are limiting factors that are expected to be tackled with adaptive evolution [6]. Additionally, understanding of how environmental conditions shape the metabolism can be enhanced through adaptive evolution. A fine-tuning of enzyme expression levels balancing the cost and burden of protein production was demonstrated by Dekel et al. [7]. The genetic basis for such short-term evolutions has been intensely studied by using genome resequencing technology [8]. However, the genetic basis of adaptations is not always obvious. For example, a rewiring of the regulatory network is reported to be a source of adaptation [9] in the tolerance of *E. coli* to ethanol. Models for evolving regulatory networks were developed by Crombach et al. [10] and Xie et al. [11]. Constraint-based models of the metabolism are already in use for predicting maximal yields of organisms and optimal outcomes of adaptive evolution [12].

Here, we present the concept of an adaptive evolution experiment in a bioreactor. In such a process, the evolutive pressure on the microorganisms for either fast growth or optimal biomass yield on a limiting substrate can be used to attain or improve the production of a desired compound. Motivated to know possible endpoints of the evolution experiment, we developed an algorithm for computing the endpoints of such an experiment. These endpoints are alternative flux distributions for the circumvention of a metabolic block. We further examined the role of a regulatory network in the usage of the alternative flux distribution and we validated the model by microarray analysis.

### Adaptive Evolution Experiment

The experiment utilized a mutant of the intestinal bacterium *Escherichia coli* which lacks both the pyruvate kinases PykA/PykF and the phosphoenolpyruvate carboxylase (Ppc). The pyruvate kinases are expected to be the main source of pyruvate on a glycerol minimal medium [13]. The Ppc reaction replenishes the tricarboxylic acid cycle (TCA) with oxaloacetate derived from phosphoenolpyruvate. It can serve as an alternative endogenous pyruvate source because oxaloacetate can be converted back to pyruvate. The Ppc reaction is reported to be an essential reaction on glycerol minimal medium [14]. As replacement for the anaplerotic reaction of Ppc the pyruvate carboxylase gene (*pyc*) of *Corynebacterium glutamicum* was inserted into the chromosomal *malEG* locus under control of the *tac*-promotor. The Pyc enzyme catalyzes the carboxylation of pyruvate to form oxaloacetate [15].

**Figure 1:**
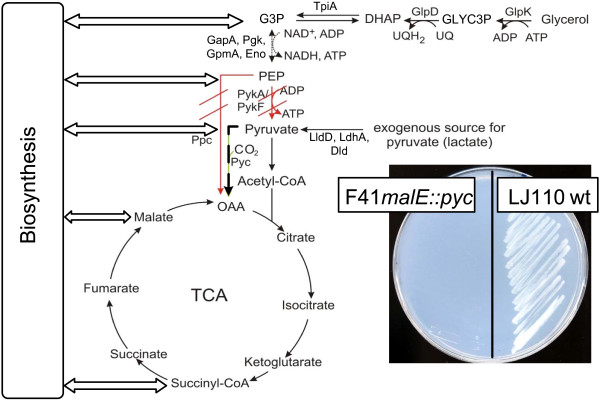
**Strain F41*malE::pyc***. The mutant F41*malE::pyc* has a deletion of the two pyruvate kinase (Pyk) genes *pykA* and *pykF*, of the phosphoenolpyruvate carboxylase (Ppc) and an insertion of pyruvate carboxylase (Pyc). On the agar plate, F41*malE::pyc* was not able to grow on glycerol minimal media and was compared to the wild type LJ110 wt. With additional lactate, F41*malE::pyc* grew. Various endogenous pathways may lead to pyruvate prototrophy.

Pyruvate is a precursor metabolite for several amino acids and also charges the TCA cycle. This is essential for the growth of the organism. Due to the knock outs, this strain F41*malE::pyc* is pyruvate-auxotrophic (see Figure 1). In contrast to our observation, Nakahigashi et al. [16] reported growth on glycerol of a *ΔppcΔpykAF* multiple mutant in their knockout study.

In the bioreactor, F41*malE::pyc* was fed with two carbon sources: Glycerol as main carbon source and lactate, which can be converted to pyruvate by one enzymatic step (Figure 1). By limiting the supply of lactate, an evolutive pressure was applied to the population in the bioreactor. Through random mutation events (e.g., in regulatory sequences of in genes encoding regulators, or in enzymes) some mutants may modify the biomass yield. Mutants that generate more biomass from the limiting substrate tend to prevail against less efficient mutants. In the experiment, adaptive evolution proceeded until the established mutant became independent from the external pyruvate source and was again pyruvate prototrophic on glycerol. The bioreactor was being operated continuously. Both the dilution rate *D*[h^−1^] and the input concentration of lactate were controlled to facilitate the prevalence of mutants with an improved yield [17].

The evolved pyruvate-prototrophic mutants had to use alternative endogenous pathways to produce pyruvate. These alternative pathways may proceed via biotechnological interesting compounds, such as the amino acids serine, or tryptophane, or as the aromatic pathway intermediate: chorismate.

**Figure 2:**
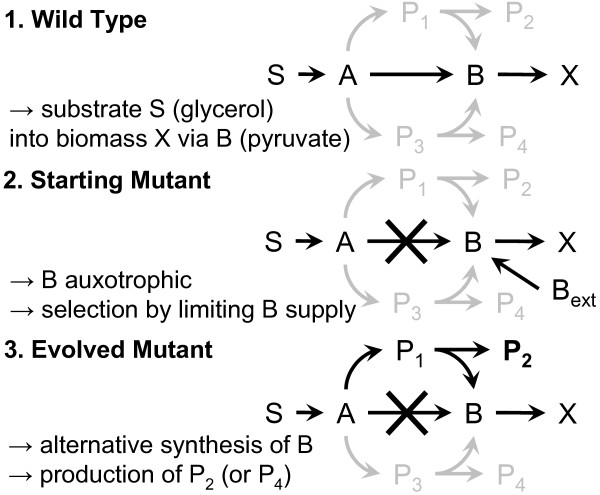
**Scheme of adaptive evolution experiment.** Simplified scheme of the strains employed and produced in the adaptive evolution experiments. Active pathways are shown in black and repressed pathways are shown in gray color.

Hence, the production of pyruvate was not the goal, but a means to attain interesting byproducts of the alternative pathways (Figure 2). In the following section, we will use a metabolic network model to explore the possibilities of evolutive adaptation [12].

## Model

The genome-scale metabolic reconstruction iAF1260 [18] contains 2077 reactions, 1039 metabolites, and additional thermodynamic information. Orth et al. [19] reviewed current flux balance analysis methods to give an overview of the possibilities of working with constraint based models. The following section analyzes the solution space of the network iAF1260 with respect to adaptive evolution.

### Constraint based model (CBM)

The metabolic compounds **C**of the network participate as reactants and products in the reactions, described by the vector of reactions $J\in R^{m}$ in [mmol h^−1^gDCW^−1^] (gDCW: gram dry cell weight). The stoichiometric information for balanced compounds was described by the matrix $N_{0}\in R^{n_{0}\times m}$ and for unbalanced compounds by $N_{e}\in R^{n_{e}\times m}$, with *n*_0_ and *n*_*e*_ as the number of balanced and unbalanced compounds, respectively. To denote the external substrate availability, the vector $b\in R^{n_{e}}$ was utilized as a boundary (e.g., if glycerol was available *b*_glyc_ was negative and if lactate was not available *b*_lac_=0). Furthermore, the growth rate was fixed to the dilution rate *J*_*μ*_ = *D* due to chemostat conditions. Since thermodynamic constraints on reactions exist, some reactions are irreversible and the direction of the flux is fixed. The following equality and inequality constraints were collected in the constraint set $K_{a}$

(1) $$\begin{array}{ll} \;0=N_{0}\;J; & \;b\leq N_{e}\;J\\ \;J_{\mu }=D; & \;J_{j}\geq 0\;\text{for some}j\;\text{(thermodynamic restrictions)} \end{array}$$

which can be further analyzed by using objective functions for optimization.

### Optimization

Properties of the constraint set $K_{a}$ of Equation (1) can be examined by applying different objective functions. A linear objective function is given by *f* = **c**^*τ*^**J**. Minimizing *f * results in an optimal value *f*_opt_ and a particular solution **J**_opt_. Applying objective functions will often result in non-unique optima. Consequently, the set of optimal solutions has to be further analyzed. By extending the constraints in Equation (1) with the equation *f*_opt_ = **c**^*τ*^**J** enforcing the optimal objective function value, a new constraint set $K_{b}$ is obtained. The set $K_{b}$ can be further analyzed by applying other objective functions.

**Yield:** The yield is defined as growth per substrate uptake $\mu \;J_{\text{up},S}^{-1}$. If the biomass is in a steady state in the chemostat, the growth rate *μ* is determined by the dilution rate *D*. For optimization purposes, the yield can be maximized by minimizing the substrate uptake *J*_up,*S*_→ min.

**Figure 3:**
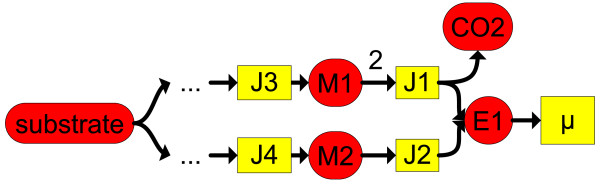
**Minimal and yield optimal minimal turnover rates.** Idea of minimal turnover rates (MTR) and yield optimal minimal turnover rates (YMTR). If *μ* > 0, the MTR via the metabolite E1 has to be greater than zero. The MTR for M1 and M2 are zero, because the paths are alternatives. If the substrate uptake rate is minimal (yield optimal), the YMTR via M2 is greater than zero. The YMTR via M1 is zero because the path via M1 is less efficient than via M2.

**Figure 4:**
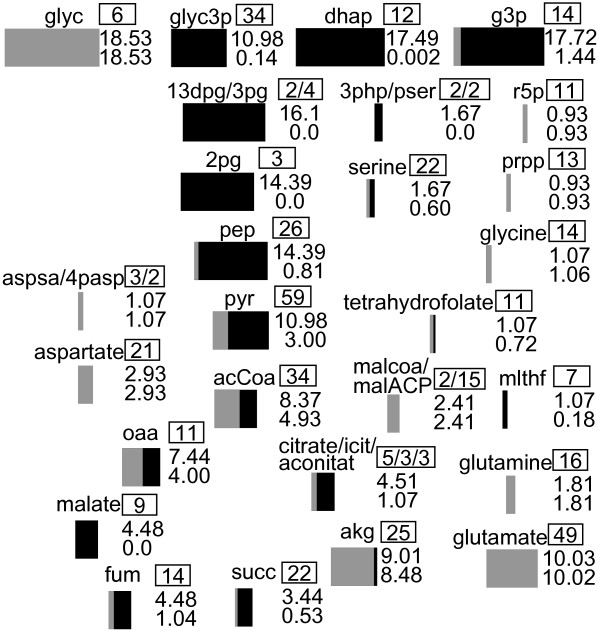
**Turnover rates.** Turnover rates of a selection of important metabolites with their number of reactions in the framed rectangle for growth on glycerol. The thickness of the black bar indicates the YMTR [mmol gDCW^−1^h^−1^] (upper number) and the gray bar denotes the MTR (lower number). The abbreviations of metabolites are presented in Section “Abbreviations”.

**Turnover rate:** With a balanced metabolite the consumption and production rate are equal, which is a measure for the turnover. We define the turnover rate as the production rate of a compound. The objective function

(2) $$\begin{array}{ll} J_{i}^{\text{MTR}}=0.5\underset{j}{\sum }|J_{j}\;\nu_{i,j}|\to \min & \; \end{array}$$

results in a minimal turnover rate (MTR) of a balanced compound *C*_*i*_ with *ν*_*i*,*j*_as a stoichiometric coefficient. A yield optimal minimal turnover rate (YMTR) was computed by extending the constraints with the fixed minimal substrate uptake rate $J_{\text{up},S}=J_{\text{up},S}^{\min}$ as outlined above and then using $J_{i}^{\text{MTR}}\to \min$ as an objective. Compounds with high turnover rates are more attractive targets for blockades in the adaptive evolution experiment, because if their main pathway is blocked the alternative pathways have to realize a high flux with potentially high formation of byproducts. The MTR were compared with YMTR in Figures 3 and 4. If the MTR is high, a blockade of these metabolites will result in a strong dependency from an external supply. If the YMTR is high compared to the MTR, the organism can improve its yield by realizing a high flux via this metabolite. Both is preferable for exerting an evolutive pressure.

### Reconstruction of alternative synthesis routes of a metabolite

**Figure 5:**
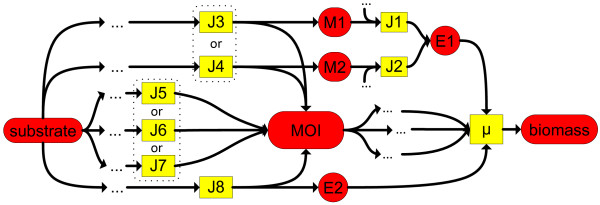
**Alternative paths to a metabolite of interest (MOI).** Illustration to identify reactions which can contribute to the production of a MOI. The metabolites E1, E2 and the MOI are essential for growth (MTR >0). For E2 there is no alternative. Thus J8 can not vary for a fixed growth rate. The alternatives (J3,J5), (J3,J6), (J3,J7), (J4,J5), (J4,J6), and (J4,J7) have to be identified.

The adaptive evolution in the experiment was based on the circumvention of a metabolic block by mutation and selection events (see Figure 2). This section presents an approach to predict pathways for the circumvention of the block. First, a method for computing combinations of reactions which are able to produce the metabolite of interest (MOI) *C*_*i*_was developed (problem illustrated in Figure 5). Second, this method was applied recursively to reconstruct alternative pathways from the external substrate to the MOI.

**1.** Determine all reactions $J_{C_{i}}$, where *C*_*i*_participates as a product. Compute their minimal rate by solving the problems $|J_{C_{i},j}|\to \min$ subject to $K_{a}$ and test with a flux-variability-analysis (FVA) [20] whether the reaction rates can vary.

**2.** Construct a constraint set $K_{C_{i}}$ by fixing all varying reactions of $J_{C_{i}}$ to their minimal rate. Let *l* be the number of constraints in $K_{C_{i}}$. $K_{C_{i}}=\left\{J_{C_{i},1}=J_{C_{i},1}^{\min},\;\ldots ,\;J_{C_{i},l}=J_{C_{i},l}^{\min}\right\}$

**3.** Starting with *k* = 1, compute the optimal yields of $\left(\begin{array}{c} \;l\\ k \end{array}\;\right)$ combinations of the joint constraints $K_{a}\cup \left(K_{C_{i}}\setminus K_{r_{k}}\right)$, where $K_{r_{k}}\subseteq K_{C_{i}}$ is a possibility to release *k* reactions. All $K_{r_{k}}$ that result in a feasible solution are joint in the set $K_{k}$.

**4.** If there is a feasible solution for the set restricting all previously found solutions $K_{a}\cup \left(\underset{\forall z\leq k}{\cup }K_{z}\right)$, increase *k* and repeat step 3.

In this manner, all minimal combinations of alternative reactions to produce _*Ci*_ were obtained. To reconstruct the alternative pathways from a substrate to the MOI, this algorithm had to be used recursively. At first it was applied to the MOI, then to the reactants of the last reactions to the MOI and then to the reactants of those reactions and so on.

The computational effort is high due to the recursive usage of the algorithm. Because the point of interest was the buildup of a metabolite’s carbon core, it was only necessary to track the reactants that carry parts of the carbon core for the metabolite. To decide whether or not a reactant contributes to the carbon core, we used the following equivalence relation:

#### Definition 1

*Given a set of cofactorsCo*, *two metabolitesC*_*i*_ and *C*_*j*_*are equivalent up toCo*( $C_{i}\overset{\mathrm{Co}}{∼}C_{j}$) if 

• a reaction *C*_*i*_ + *Co*_*k*_→*C*_*j*_or*C*_*i*_→*C*_*j*_ + *Co*_*k*_exists, or

a metabolite *C*_*x*_exists, for that is known, that $C_{i}\overset{\mathrm{Co}}{∼}C_{x}\wedge C_{j}\overset{\mathrm{Co}}{∼}C_{x}$, or

a reaction *C*_*i*_ + *C*_*x*_→*C*_*j*_ + *C*_*y*_with $C_{x}\overset{\mathrm{Co}}{∼}C_{y}$ exists.

Those cofactors were chosen as H^+ ^, HO_4_P, NH_4_, H_2_O. For example, ATP, ADP, and AMP are equivalent up to these cofactors.

Furthermore, the production of many metabolites was possible via alternative end reactions but amounted to the same precursor metabolites. This fact also reduced the computational effort, because the multiple evaluation of common precursors was avoided.

The recursive application of the algorithm is an alternative approach for computing the elementary modes in this special task. Computing the elementary modes for a model like iAF1260 would be an extreme computing-intensive task, even if methods for network compression are used [21]. The algorithm proposed above reduces the computational effort by excluding some reactants as a source for the carbon core of a metabolite.

#### Evaluation for F41*malE::pyc*

In the experiment (see Figure 2) pyruvate was chosen as the metabolite of interest. In the model iAF1260, pyruvate appeared in 59 reactions as a reactant, it had a high MTR and YMTR (Figure 4) and several alternative synthesis routes are known. Hence a metabolic block of pyruvate formation seemed suitable to study adaptive evolution.

**Figure 6:**
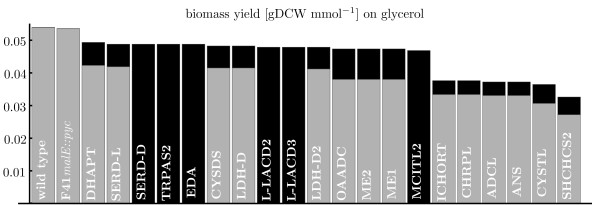
**Yields for alternative pyruvate formation.** Possible reactions to produce pyruvate (sorted by model predicted biomass yield on glycerol) computed by the proposed algorithm. The black bars show the predicted biomass yields on glycerol without regulation, gray bars with the regulatory network iMC1010v2. The bars for wild type and F41*malE::pyc* use combinations of reactions to pyruvate. Other bars are tagged by their final reaction to pyruvate: DHAPT - Dihydroxyacetone phosphotransferase (DhaK, DhaL, DhaM), SERD-L - L-Serine deaminase (TdcG, SdaA, SdaB, TnaA), SERD-D - D-Serine deaminase (DsdA), TRPAS2 - Tryptophanase-L-tryptophan (TnaA), EDA - 2-Dehydro-3-deoxyphosphogluconate aldolase (Eda), CYSDS - Cysteine desulfhydrase (TnaA, MetC), LDH-D - D-lactate dehydrogenase (Ldh), LACD2 - L-lactate dehydrogenase using ubiquinone (LldD), LACD3 - L-lactate dehydrogenase using menaquinone (LldD), LDH-D2 - D-lactate dehydrogenase (Dld), OAADC - Oxaloacetate decarboxylase (Eda), ME2 - Malic enzyme NADP (MaeB), ME1 - Malic enzyme NAD (MaeA), MCITL2 - Methylisocitrate lyase (PrpB), ICHORT - Isochorismatase (EntB), CHRPL - Chorismate pyruvate-lyase (UbiC), ADCL - 4-aminobenzoate synthase (PabC), ANS - Anthranilate synthase (TrpD, TrpE), CYSTL - Cystathionine-b-lyase (MalY, MetC), SHCHCS2 - 2-Succinyl-6-hydroxy-2-4-cyclohexadiene-1-carboxylate synthase (MenD). In all cases either ALATA-L - Alanine transaminase (AlaABC) (shown yields) or DXPS - Deoxy-D-xylulose-5-phosphate synthase (Dxs) contributed to pyruvate production similar to J3 and J4 in Figure 5. Other essential reactions similar to J8 in Figure 5 were: ACLS - Acetolactate synthase (IlvH or IlvB), ACHBS - 2-Aceto-2-hydroxybutanoate synthase (IlvH or IlvB) and DHDPS - Dihydrodipicolinate synthase (DapA).

**Figure 7:**
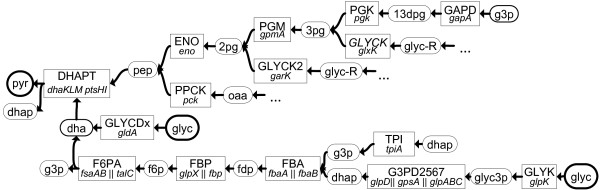
**Alternative paths via DHAPT.** Alternative paths from glycerol to pyruvate via dihydroxyacetone kinase (DHAPT). The abbreviations of metabolites are presented in Section “Abbreviations”.

**Figure 8:**
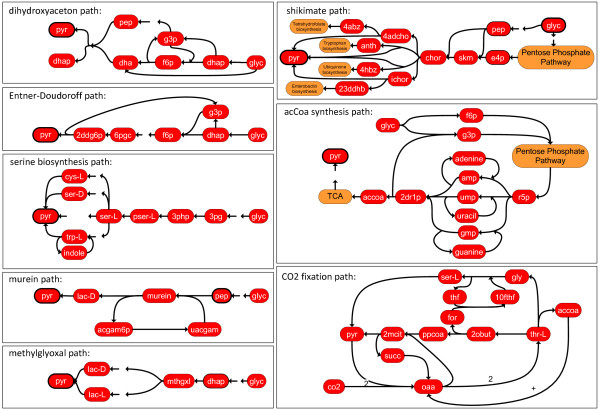
**Alternative pathway classes.** Illustration of the eight alternative pathway classes to pyruvate. The abbreviations of metabolites are presented in Section “Abbreviations”.

The above algorithm was employed to reconstruct alternative pathways from the carbon source glycerol to pyruvate. With *k* = 2, all minimal combinations of reactions to produce pyruvate were obtained (results see Figure 6). The recursive usage of the algorithm above resulted in a variety of flux distributions that represented alternative pathways from glycerol to pyruvate, which were summarized in flux maps. Several flux distributions utilized the same precursors but differed in an alternative reaction from one precursor to another. The flux distributions were categorized using key metabolites and manual post-processing (see Figure 7 and Additional file 1: Table S1). In this manner, eight alternative pathway classes were found (see Table 1 and Figure 8).

**Table 1:** Alternative pathway classes from glycerol to pyruvate

**Class**	**Characteristics**	**Max.**	**TFN**	**Microarray**	
		**yield**	**prediction**	**LJ110 /**	**F41 *****malE::pyc /***
				**K98-62**	**K98-62**
dihydroxyacetone-	dihydroxyaceton (toxic), *dhaKLM* operon is	0.0404	active	active	active
path	controlled by DhaR		(includes		
			no DhaR)		
Entner-Doudoroff-	*eda* and *edd* controlled by GntR	0.0365	repressed	active	active
path					
serine biosynthesis	transamination step from 3-phosphoglycerate	0.0384	active	active	active
	to serine, various degenerating paths to		(via L-serine)		
	pyruvate via L-serine, D-serine, L-cysteine				
	and L-tryptophane				
shikimate path	generates chorismate, pyruvate occurs as	0.0214	active	active	active
	a byproduct for tryptophan-, enterobactin-,				
	tetrahydrofolate-, ubi/menaquinone-				
	biosynthesis; secretion of enterobactin possible				
methylglyoxal path	methylglyoxal (toxic) is formed from dhap	0.0365	active	active	active
	and detoxified in 3 different ways to lactate				
acCoA synthesis	utilize deoxyribonucleotides as carbon shuttle	0.0344	repressed	active	active
	AMP, UMP and GMP are synthesized and				
	degraded				
murein path	via synthesis and degradation of murein	0.0297	repressed	(active)	active
CO_2_ fixation	2 pyruvate are reinvested to form	0.0269	active	active	active
	2 oxaloacetate; carbon transfer between				
	glycolysis/pentose phosphate pathway and				
	TCA occurs only via CO_2_				

^a^The yields [gDCW mmol^−1^] on glycerol were calculated for the experimentally determined maximal growth rate of the evolved strain K98-62 (*μ* = 0.25 h^−1^). To compute the maximal yield for a single alternative pathway (AP) all other APs were restricted to their minimal value. For the prediction of the TFN, the environmental conditions of the chemostat (MM with glycerol) were used as an input. The APs have several important reactions for generating a yield. If the average expression level of genes for enzymes of those important reactions drops below a threshold of 7.0 units, we assumed that the enzymatic capacity to perform the reaction is not present.

After performing multiple independent evolution experiments with the pyruvate-auxotrophic mutant F41*malE::pyc* (see Figure 1), a total of five pyruvate-prototrophic strains with different characteristics were isolated after 200–300 generations each. One of the strains (K98-62) showed an increased enterobactin secretion. This property was part of the predicted shikimate pathway class (Figure 8). The strain was exposed to different media where pyruvate prototrophy had no growth benefits and the phenotype remained stable. This indicated that the pyruvate prototrophy was caused by a change of the genotype. A sequencing of the whole genome showed about 400 changes (including three deletions) compared to the wild type W3110 [22], but none of them has been yet assigned to be decisive (most mutated genes were prophage genes, operative genes with high probability of mutation were explored in detail). The genotypic alterations do not give an obvious explanation for the phenotype of the evolved strain, e.g., the enterobactin secretion. In order to clarify the relation between genotype and phenotype of the evolved strains, a transcriptional network model was studied.

### Impact of transcriptional regulation

The transcriptional network of an organism can be interpreted as an information processing unit for the cell transmitting signals from the environment to enzyme availabilities via gene transcription [23]. Transcriptional regulation avoids production of enzymes which are unprofitable under certain environmental conditions. This contributes to evolutionary fitness. However, a tradeoff exists between fitness advantage due to reduced protein cost and reduced response time after a change of environmental conditions [24].

After a directed genetic change of the organism (e.g., a knockout of pyruvate kinase), the regulatory network is not necessarily optimal any more. Consequently, random mutations leading to an altered regulatory network were expected by Crombach et al. [10] as a driving force for adaptive evolution.

We intended to study how transcriptional regulation affects the availability of enzymes that are essential for the predicted alternative pathways (Table 1). Therefore, the Boolean transcription factor network (TFN) iMC1010v2 [25] with 104 regulatory genes and an influence on 479 genes was adapted to the metabolic network iAF1260 (see Additional file 2: Table S2). The model provided Boolean formulas that describe how environmental conditions act on the gene expression via the transcriptional regulatory network. The TFN had no feedback loops [26]. For this reason, variables describing environmental conditions could be used as an input of the TFN and a unique Boolean steady state was achieved. As the Boolean steady state describes on/off gene activities, these were translated via gene-protein-reaction associations of iAF1260 in reaction constraints. We assumed that a flux can not occur if genes were off that code for a respective enzyme. The proposed “off” genes extended the set of constraints for the optimization problem in Equation (1). The transcriptional constraints reduce the solution space of the metabolic model. Assuming a fixed biomass composition, the predicted biomass yield of the metabolic model without such constraints is greater than or equal to those with additional transcriptional constraints. We analyzed, as a first step, the predictive power of the combination of metabolic network (MN) and TFN.

#### Analysis of the metabolic and transcriptional model

**Figure 9:**
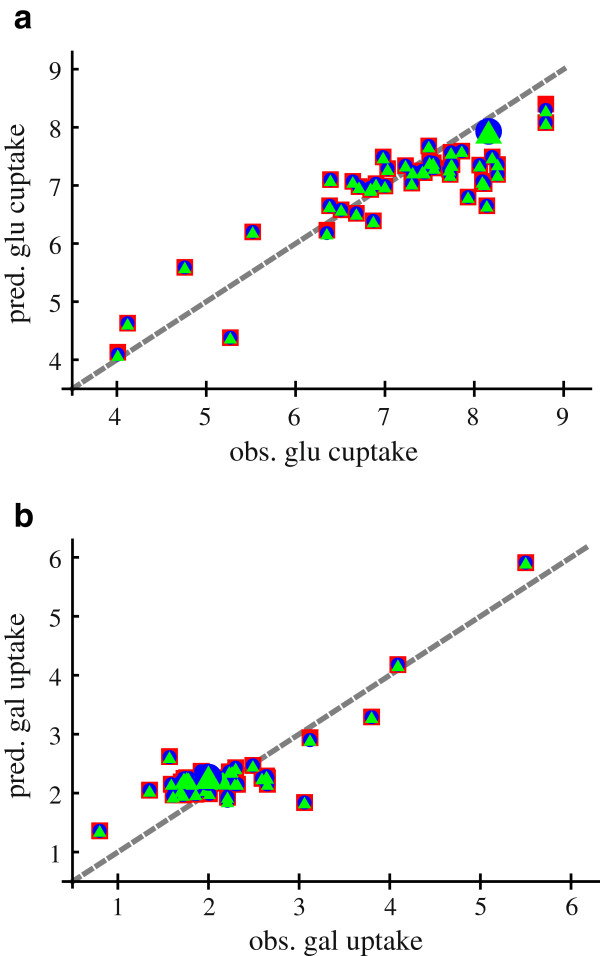
**Predictive efficiency of metabolic network (MN) and transcriptional network (TN).** Predictive efficiency of metabolic network (MN) and transcription factor network (TFN). Shown is the measured glucose **(a)**/ galactose**(b)** consumption in [mmol gDCW^−1^h^−1^ of the transcription factor knockout study of [27] versus the predicted consumption. The measured growth rate and acetate secretion of the study are used to predict the glucose/galactose uptake. The green triangles show predictions of the MN alone, the blue dots show predictions of the TFN combined with MN and the red squares include the knockouts of the study in the TFN combined with MN. The big green and blue dots show the values of the wild type. Statistical analysis of the model: observed uptake rate ≈ predicted uptake rate, results in an estimated error variance of 0.2811 for the MN, 0.2816 for MN with TFN and 0.2812 for MN with TFN and knockout.

We used data of the transcription factor knockout study of Haverkorn et al. [27] in order to analyze the predictive effectiveness of the metabolic model restricted by the transcriptional model. This study contains measurements of specific growth rates, specific acetate secretion rates and substrate uptake rates for glucose and galactose for 81 transcription factor and 10 *σ*and anti-*σ*factor knockouts. Only 41 of the evaluated factors are included in the iMC1010v2 model. The environmental conditions of the experiments were expressed in a constraint set such as $K_{a}$ of Equation (1) and extended by a constraint for the measured acetate secretion rate and the measured growth rate to the constraint set of the MN $K_{m}$. The environmental conditions of the experiment were used as an input of the TFN iMC1010v2 and resulted in the constraint set $K_{\text{tf}}$. The knockouts of transcription- or *σ* factors changed the TFN and resulted in a changed constraint set $K_{\text{tf},-k}$, where *k* denoted the factor which was deleted. The objective function of minimal substrate uptake was evaluated on the constraint sets $K_{m}$, $K_{m}\cup K_{\text{tf}}$, and $K_{m}\cup K_{\text{tf},-k}$ yielding three predictions of substrate uptake, which were then compared with the measured substrate uptake. The outcome of the comparison is shown in Figure 9. The analysis revealed that there was no improvement of the prediction of the metabolic model through the extension with the transcriptional model. However, this result should be interpreted with care. First, in case the observed uptake rate was lower than the predicted uptake rate of the MN, the model extended by the TFN had to result in an equal or even worse prediction, because the TFN additionally restricted the solution space of the MN. Second, if a transcription factor in reality has no impact on the substrate yield, the prediction of the MN should be equal to the TFN extended MN, which seemed to be the case in most predictions. Third, if the TFN is partially incomplete, the prediction tends to be conservative and does not restrict the reaction fluxes. Under conditions of aerobic growth on glucose/galactose (conditions of the study of Haverkorn et al. [27]) the TFN had a low impact on the substrate uptake. Therefore, no real assessment of the quality of the TFN can be made. With this limitation in mind we present here the analysis of an evolutionary trajectory of F41*malE::pyc*.

#### Analysis for F41*malE::pyc* and its evolved strains

The wild type strain LJ110 (W3110) [28], the strain F41*malE::pyc* and the evolved strains showed different growth features. In fact eight alternative metabolic pathways exist to circumvent the metabolic block of regular pyruvate formation. This raised the question why F41*malE::pyc* was not able to use these eight alternative metabolic pathways on minimal media (MM) with glycerol. After a cultivation phase of F41*malE::pyc* in MM with glycerol, growth could be obtained by adding lactate. This indicated that F41*malE::pyc* was not poisoned by toxic metabolites. The useability of some of the eight alternative pathways was proven by the sheer existence of the evolved pyruvate prototrophic strains. Therefore, we examined the hypothesis if transcriptional regulation prevents the transcription of genes which are essential for the usage of the alternative pathways. We checked how the transcriptional network impacts the eight alternative pathways (Table 1) and validated the enzymatic capabilities of the strains by transcript analysis after growth in different minimal media. The alternative metabolic pathways were employed to identify important reactions for pathway functionality. The gene-protein-reaction associations were used to conclude which genes needed to be transcribed in order to ensure a reaction flux for functionality of the pathways. To decide whether the enzymatic capacity for catalyzing a reaction was available, a threshold for the average measured expression was used, neglecting regulation on a post-transcriptional level. For the evaluation we chose a threshold of 7.0. The enzymatic capacity necessary for functionality of the eight alternative pathways as determined by mRNA measurements was summarized in Table 1 (detailed information see Additional file 1: Table S1) together with the predicted restrictions by the TFN. The wild type and K98-62 were compared on MM with glycerol; and F41*malE::pyc* and K98-62 were compared on MM with glycerol and lactate. The analysis of the differential expression values revealed no clear indication for an up/down regulation of a metabolic pathway. This statement is based on the following two findings which can be drawn from Additional file 1: Table S1. First, all the genes considered, were being transcribed under the given conditions. Thus, their gene product may contribute to pyruvate delivering pathways. Second, the absence of gross changes in transcripts did not provide hints towards regulatory changes which would explain a direct assignment to a pathway. This makes it more likely that the activity of some enzymes may be altered due to metabolic feedbacks. We looked for other systematically up/down regulated genes and found that genes associated with iron-sulfur cluster assembly were upregulated in the evolved strain.

The results indicated that estimating from the microarray data, the enzymatic capability of using the alternative pathways was available in all three compared strains. The TFN did not predict a transcriptional downregulation of the pathways in most cases, which was in accordance with the microarray data, except for the Entner-Doudoroff-path and alternative acetyl-CoA synthesis path. This means that the hypothesis of restricting the alternative pathways by transcriptional regulation did not hold.

## Conclusion

We have illustrated the idea of an adaptive evolution experiment in a chemostat bioreactor, where mutation and selection led to circumvention of a metabolic block. Constraint-based methods were utilized to identify targets for blocks and to predict alternative pathways for this circumvention. We performed the experiment with a pyruvate-auxotrophic strain F41*malE::pyc* on glycerol with an additional pyruvate source. The introduced algorithm for the computation of alternative pathways was employed to predict pathways from glycerol to pyruvate as possible endpoints of evolution for the strain F41*malE::pyc*. The evolution experiment with F41*malE::pyc* resulted in five evolved strains. This proved that the usage of alternative pathways was possible after adaptive evolution. However, F41*malE::pyc* was unable to grow without a pyruvate source. We assumed that transcriptional restriction of the predicted pathways hindered the growth. Therefore, a Boolean transcription factor network (TFN) was employed to further restrict the solutions of the metabolic network (MN). The prediction of the TFN together with microarray analysis revealed that in this case it is improbable that transcriptional regulation was exclusively responsible for the activation of the proposed alternative pathways during adaptation. It was shown that mRNA of genes which are important for the functionality of the predicted pathways were present in all compared strains.

However, a clear elucidation of the course of genetic events during adaptation was not yet possible. Metabolic feedbacks and non-regulatory effects may play an important role. We believe that the TFN will help to support further analysis by giving the possibility to determine the regulatory effects of metabolic and environmental signals and to distinguish between cause and effect of the up/down regulation of a gene. This will warrant further study in the field of transcription factor networks and their input in order to understand the whole sequence of events during adaptive evolution.

## Materials and methods

### Strains, medium, and growth conditions

The strains used in the experiments are listed in Table 2. The minimal medium (MM) (modified after [29]) used for all experiments consisted of 4.7 g NaH_2_PO_4_·2 H_2_O, 11.5 g K_2_PO_4_, 2.64 g (NH_4_)_2_SO_4_, 0.74 g MgSO_4_·7H_2_O, 14.7 mg CaCl_2_·2H_2_O, 13.5 mg ZnCl_2_, 2.8 mg FeSO_4_·7H_2_O, 10*μ*l 1N HCl, 20 mg Thiamine, 0.2 mM IPTG per liter. In shaking flask experiments 0.5% (w/v) glycerol was used as carbon source. When a mixture of glycerol and D,L-lactate was used the concentrations were 0.4% glycerol and 0.1% DL-lactate in order to have constant carbon availability in all experiments. Cells were cultivated in 250 ml shaking flasks filled with 25 ml growth medium. Prior to use, the cells were streaked onto LB-agar plates freshly from −80°C frozen stocks and incubated overnight at 37°C. Single colonies from LB-agarplates were then adapted to growth on minimal medium on MM agar plates for three days. Cultures were initiated directly from MM agar plates at OD600 = 0.1. After overnight incubation at 37°C without shaking the cells were grown at 37°C and 70 rpm.

**Table 2:** Strains

**Strain**	**Genetic properties**
W3110 (LJ110)	^F−^^*λ*−^*rpoS*(Am) *rph*-1 Inv (*rrnD-rrnE*) ( [30]; [22])
F41*malE::pyc*	LJ110 *ΔppcΔpykAΔpykFΔmalE*−*G*::*P*_tac_−^*pyc* + ^[17]
K98-62	Evolved from F41*malE::pyc* during longterm cultivation in a chemostat on minimal
	medium with glycerol as carbon source; pyruvate-prototrophic (this study)

### Bioreactor

Chemostat fermentations were performed in a Bioengineering fermentor KLF at 37°C, with stirring rate of 500–1000 rpm, an input air of 1 L/min, controlled pH at 7 and pO_2_ was kept above 50%. The feeding contained MM with 5 g/L glycerol and lactate concentrations in the range from 0.125 g/L down to 0 g/L of 95% L-lactate. The last 100 fermentation hours the feeding contained no lactate. The glycerol concentration measured in the fermentation broth was close to zero.

### Genome resequencing

For genome resequencing the cells were grown to stationary phase. Chromosomal DNA was isolated via Phenol/Chloroform precipitation [31]. The resequencing was performed by LGC Genomics (Berlin, Germany) using 454 FLX Titanium Sequencing. The sequence of K98-62 was mapped to the online available sequence of E. coli W3110 (AP009048.1).

### Transcriptome analysis

For transcriptome analysis strains K98-62 and LJ110 were compared after growth on minimal medium containing glycerol as carbon source and K98-62 and F41*malE::pyc* were compared after growth on minimal medium containing glycerol and D,L-lactate as carbon source. Cells were harvested after reaching OD600 = 0.8. The DNA chips were custom-synthesized by Agilent company and processed according to the manufacture’s instruction. A complete description of transcript data will be published elsewhere, but can be obtained from the authors directly.

The average expression is the mean value of all normalized Log_2_ spot intensities over all biological replicates and colors. If the average expression value of a mRNA was measured below 6 units, it is uncertain that the mRNA was present in the probe. If the fold change value was not significantly different from zero and the average expression value was above 7.0, we assumed that mRNA of a gene was present in both compared strains. Observing significant fold change values, we studied the strain specific average expression to assess whether mRNA was present or not. The mean average expression value over all spots was 6.93 in the comparison K98-62 versus wild type and 7.37 for K98-62 versus F41*malE::pyc*. Data to estimate the enzymatic capability of the predicted pathways was included in Additional file 1: Table S1.

### Constraint based model analysis

In Equation (1), we regarded also ATP requirements for the maintenance metabolism *J*_ATPm_ in $K_{a}$. Although the maintenance metabolism may vary on different substrates and in the evolved strains, we decided to fix the rate of this flux for the computations and therefore the value for an aerobic culture on glucose *J*_ATPm_ = 8.39[mmol^h−1^^gDCW−1^ from Feist et al. [18], which is a theoretical calculation. This value, however, does not influence the structure of the identified pathways, but it has an impact on the yield numbers in Figures 4 and 6.

Combination of iAF1260 and iMC1010v2: The computation of a regulatory model combined with metabolic model was outlined by Covert et al. [32]. The network iMC1010v2 was originally designed for the metabolic network iJR904 [33]. The model extension iAF1260 has a much more detailed reaction of biomass formation. Hence, some reactions became essential due to model extension, but were downregulated by the iMC10010v2. We identified these reactions and made these independent from the Boolean regulatory model. Details are shown in Additional file 2: Table S2.

## Abbreviations

accoa: Acetyl-CoA; prpp: 5-Phospho-α-D-ribose-1-diphosphate; acgam6p: N-Acetyl-Dglucosamine6phosphate; pser: Phospho-L-serine; akg: Oxoglutarate; pyr: Pyruvate; anth: Anthranilate; r5p: α-D-Ribose-5-phosphate; aspsa: L-Aspartate-4-semialdehyde; ser-D/L: D/L-Serine; chor: Chorismate; skm: Shikimate; cys-L: L-Cysteine; succ: Succinate; dha: Dihydroxyacetone; thf: Tetrahydrofolate; dhap: Dihydroxyacetone-phosphate; thr-L: L-Threonine; e4p: D-Erythrose-4-phosphate; trp-L: L-Tryptophan; fdp: D-Fructose-1-6-bisphosphate; uacgam: UDP-N-Acetyl-D-glucosamine; for: Formate; 10fthf: 10-Formyltetrahydrofolate; fum: Fumarate; 13dpg: 3-Phospho-D-glyceroyl-phosphate; f6p: D-Fructose-6-phosphate; 2ddg6p: 2-Dehydro3deoxy-D-gluconate6phosphate; glyc: Glycerol; 2dr1p: 2-Deoxy-D-ribose-1-phosphate; glyc-R: R-Glycerate; 2mcit: 2-Methylcitrate; glyc3p: Glycerol-3-phosphate; 2obut: 2-Oxobutanoate; g3p: Glyceraldehyde-3-phosphate; 2pg: D-Glycerate-2-phosphate; ichor: Isochorismate; 23ddhb: 2-3-Dihydro-2-3-dihydroxybenzoate; lac-D/L: D/L-Lactate; 3pg: 3-Phospho-D-glycerate; malACP: Malonyl-acyl-carrier-protein; 3php: 3-Phosphohydroxypyruvate; malcoa: Malonyl-CoA; 4abz: 4-Aminobenzoate; mlthf: 5-10-Methylenetetrahydrofolate; 4adcho: 4-Amino-4-deoxychorismate; mthgxl: Methylglyoxal; 4hbz: 4-Hydroxybenzoate; oaa: Oxaloacetate; 4pasp: 4-Phospho-L-aspartate; 6pgc: 6-Phospho-D-gluconate.

## Competing interests

The authors declare that they have no competing interests.

## Authors’ contributions

RF has written the manuscript, has done modeling and experimental study and did conceptual study. KG has done experimental study including transcript analysis. GV has done the computations for the model analysis of transcription factor knockout study. JK and SS have supervised the study of GV and did theoretical studies with the transcriptional network. MB and OS contributed to the design of the project. GS contributed to the design of the project and to the interpretation of data. ME contributed to the design of the project, has developed a preliminary version of the computational algorithm, has done conceptual study on the article and helped to interpret the data. All authors read and approved the final manuscript.

## Supplementary Material

Additional file 1 Alternative pathway classes. Additional file 1 contains detailed information about the alternative pathway classes from Section “Abbreviations”. click here for file

Additional file 2 Modifications of iMC1010v2, input signals and predicted inactive genes. Additional file 2 describes how iMC1010v2 can be adapted to the metabolic model iAF1260. It contains input signals for three environmental conditions and the according outputs. click here for file
